# Transferring 24/7 sobriety from South Dakota to South London: the case of MOPAC's Alcohol Abstinence Monitoring Requirement Pilot

**DOI:** 10.1111/add.14609

**Published:** 2019-04-23

**Authors:** Laura Bainbridge

**Affiliations:** ^1^ Department of Social Policy London School of Economics and Political Science London UK

**Keywords:** Alcohol Abstinence Monitoring Requirement, compulsory sobriety, elite interviewing, policy transfer, South Dakota 24/7 Sobriety Project, subnational policymaking, violence reduction

## Abstract

**Background and aims:**

During the past three decades an expansive literature has emerged that is dedicated to analysing the processes of policy transfer. One neglected pathway involves subnational agents emulating crime control innovations that have emerged in subnational jurisdictions of other nations. This paper presents the case of the London Mayor's Office for Policing and Crime's (MOPAC) Alcohol Abstinence Monitoring Requirement (AAMR) Pilot to examine the multi‐level factors that facilitate and/or constrain international–subnational crime and justice policy transfer.

**Methods:**

A qualitative case study design reconstructed the (in)formal events that led to components of the South Dakota 24/7 Sobriety Project (USA) being either abandoned or integrated into MOPAC's AAMR Pilot. Evidence is drawn from elite interviews and documentary materials.

**Results:**

A series of inter/transnational‐, macro‐domestic‐, meso‐ and micro‐level factors enabled and/or obstructed processes of complete international–subnational policy transfer. Exclusion of domestic violence perpetrators from the London Pilot was fuelled by interest‐group hostility and mobilization. Use of alcohol tags rather than breathalysers to monitor compliance was a result of political–economic constraints, concern surrounding intrusion, technological innovation and policy‐orientated learning. The decision to omit an ‘offender pays’ funding mechanism was a consequence of legal incompatibility and civil service reluctance, while ‘flash incarceration’ for breach was not implemented due to European policy harmonization.

**Conclusions:**

The London Alcohol Abstinence Monitoring Requirement Pilot was a policy ‘synthesis’ that combined ideas, goals, vocabulary, principles, technology and practices from the South Dakota model with the existing English and Welsh criminal justice framework. Structural factors and the actions of particular agents limited the extent to which policy transfer occurred.

## Introduction

The claim that crime control policies ‘travel’ is commonplace, but one that is rarely supported by empirical evidence. Indeed, despite assertions that a plethora of foreign innovations have been borrowed by policymakers in the United Kingdom, few studies have been conducted that directly examine the occurrence and realities of crime and justice policy transfer, defined here as:

A process in which knowledge about crime and justice institutions, policies or delivery systems in one jurisdiction is used in the development of crime and justice institutions, policies or delivery systems in another jurisdiction in a different country (definition adapted from 1).

Instead, hunches pertaining to the overseas origins of policy initiatives such as drug courts, mandatory drug testing for arrestees, restorative justice, day fines, problem‐orientated policing, civil gang injunctions, boot camps, Neighbourhood Watch, Ugly Mugs and street wardens generally prevail. Those studies that have been published have primarily focused on the national level, and explore associations and similarities between crime control policy in the United Kingdom and the United States; see 2, 3, 4, 5, 6. Focus on the United States has largely been a consequence of, and contributor to, heightened criminological interest in the ‘Americanization’ of UK policymaking and the associated convergence and divergence in crime control debate—that is, a debate that concerns the extent, causes and implications of globalization with regard to criminal justice and penal policy 4. Arguably, however, persistent focus on national‐level developments and neglect of the phenomenon of international–subnational crime and justice policy transfer is problematic for two reasons. First, policy change can, and does, emerge in a bottom‐up rather than top‐down fashion, with subnational agents such as those associated with local or regional administrations cultivating interventions that are subsequently adopted by central government or that diffuse horizontally across localities 5, 7, 8, 9, 10, 11. Secondly, the police accountability and governance reforms introduced by the UK Coalition Government (2010–15) have ostensibly created a new opportunity structure for crime and justice policy transfer to occur by placing directly elected Police and Crime Commissioners (PCCs) into a subnational strategic leadership position. Given that PCCs may prove to be key agents of policy emulation over time 12, questions pertaining to the processes of international–subnational crime and justice policy transfer arguably require answers. In analysing the case of the London Mayor's Office for Policing and Crime's (MOPAC) Alcohol Abstinence Monitoring Requirement (AAMR) Pilot, this paper takes steps to address one such question: specifically, what factors facilitate and/or constrain the adoption and implementation of an overseas subnational crime and justice innovation in the United Kingdom? It will be argued that a series of multi‐level factors enabled and/or hindered the transfer of the 24/7 Sobriety Project from South Dakota to London, leading to some elements of the project being emulated and others being adapted or abandoned.

## The South Dakota 24/7 Sobriety Project and MOPAC's AAMR pilot

In 2008, Kit Malthouse was appointed by the Mayor of London, Boris Johnson, to be his Deputy Mayor for Policing. At this time, two strands of interpersonal violence in London appeared to be bucking national crime trends by not declining: violence that was occurring within the night‐time economy and domestic violence 13. In searching for a solution, Malthouse attended the eighth Oxford Policing Policy Forum (OPPF). This Forum was established in 2006 as a joint initiative of the Centre for Criminology at the University of Oxford and the independent think‐tank, the Police Foundation. Its purpose is to provide a safe space for senior stakeholders to network and to have an informal and energetic ‘warts‐and‐all’ debate under Chatham House Rules about policing issues 14. Participation is via direct invitation only. In light of the increase in drug‐ and alcohol‐related crime in the United Kingdom and the reactive publication of a drug law enforcement strategy developed by the now disbanded UK Drug Policy Commission, the theme of the OPPF attended by Malthouse was *Policing drugs and alcohol: is harm reduction the way forward?* 14. It was during this event that he was introduced to the South Dakota 24/7 Sobriety Project by Professor Jonathan P. Caulkins (Carnegie Mellon University). In delivering a presentation at the outset of the OPPF, Professor Caulkins included a succinct section that was dedicated to outlining ‘24/7 Sobriety’, along with a series of descriptive statistics pertaining to the efficacy of this coerced alcohol abstinence strategy 14, 15.

24/7 Sobriety emerged in Bennett County, a rural jurisdiction in South Dakota, United States. The County's prosecutor, Larry Long, identified that almost every crime committed in his region was linked to alcohol, and that the same individuals were being repeatedly sentenced and released from jail 16. To alleviate this revolving‐door situation, Long devised an approach to address alcohol‐related crime and recidivism, in particular drink‐driving and domestic violence. The idea was simple. Defendants who had demonstrated that their alcohol intake was a threat to public safety would have their ‘licence to drink’ suspended in the same way that those who fail to operate a vehicle responsibly have their driving licence revoked 17. With cooperation from a local judge, Long's project launched in 1985. As a condition of bond and until their cases were resolved, defendants were required to present themselves twice daily, 7 days a week, at a sheriff's office and undertake a breath test for a reading of their blood–alcohol concentration levels. Those who tested positive (i.e. ‘blew hot’) or who failed to show up for a scheduled test (i.e. ‘no shows’) were ‘flash incarcerated’ in the county jail, typically for a few days. The initial results of Long's method were considered encouraging. Not only did individuals attend as required, but a high proportion blew negative tests, including some deemed to be alcohol‐dependent 18. Moreover, the jail population decreased and drink‐driving and domestic violence rates declined across the county 19.

Long relocated to Pierre (the state capital of South Dakota) in 1991 and was later elected Attorney General. In 2003/4 he was appointed to a task‐force charged with reducing the state prison population. Having recognized that substance misuse continued to lie behind much of the work of the criminal justice system, he suggested that an initiative similar to his Bennett County project be piloted. The 24/7 Sobriety Project began in February 2005 in three counties. The pilot initially targeted drink‐driving defendants with at least one prior drink‐driving conviction within the previous 10 years. Software was developed to track the results of each defendant's test data, and participants helped to support the cost of the initiative by paying $1 per test. Judges in the pilot jurisdictions considered 24/7 Sobriety to be a success and, via word of mouth, the project began diffusing to other South Dakota counties 17. Expansion of the project, however, presented a number of design challenges. Some defendants had to drive up to 50 miles to be tested because they lived or worked in a rural location that did not have a local jail or sufficient law enforcement personnel to administer the breathalysers 16, 18. Other defendants worked unusual hours or had transport issues, which made it burdensome for them to report to the test site on time. To resolve these difficulties, those defendants for whom breath testing was unfeasible were issued with transdermal alcohol monitoring devices (alcohol tags). An additional complication encountered was that while intensive monitoring was keeping defendants sober, some were opting to use illegal and prescription drugs as an alcohol substitute 16, 19. As a result, sweat patches and random urinal testing were introduced into the project. In 2007 the South Dakota legislature unanimously approved the creation of a state‐wide 24/7 Sobriety programme administered by the Attorney General's Office. The legislation permitted the use of 24/7 Sobriety conditions for all crimes in which alcohol and/or drugs played a role in their commission, and widened eligibility to incorporate probationers and parolees as part of their supervision. The 2007 legislation similarly modified state law to permit juvenile court judges involved in abuse or neglect cases to enrol caregivers into the 24/7 Sobriety testing regime as a condition of returning children to their home.

Following his attendance at the OPPF, Malthouse instructed his team within the Greater London Authority to undertake desk research into the architecture and outcomes of 24/7 Sobriety. Then, in late 2010, he commenced a London compulsory sobriety pilot campaign. At the heart of his vision were the core components of the South Dakota model: court‐mandated alcohol abstinence; regular monitoring; offender pays; and ‘swift, certain and fair’ punishment for breach. Domestic abusers were highlighted as potential pilot participants, along with drink‐drivers and night‐time economy offenders. Four years later, under the leadership of Malthouse's successor, Stephen Greenhalgh, MOPAC launched an AAMR Pilot in the South London Local Justice Area. As illustrated in Table 1, the structure of this pilot was somewhat different to that proposed previously. Compulsory sobriety was not utilized as a post‐release license condition; the AAMR period was shorter than that initially proposed; alcohol tags rather than breathalysers were adopted to ensure compliance; and offender pays and ‘swift, certain and fair’ punishment for violations were omitted. In addition, domestic violence perpetrators were excluded from participating.

**Table 1:** The London ‘Compulsory Sobriety’ Pilot.

Proposed, 2010	Implemented, 2014
Criminal justice sentence	
• Community Order or post‐release licence condition • Duration: 1–2 years (depending on compliance)	• Community or Suspended Order: punitive requirement. The AAMR can standalone or can be combined with other requirements • Duration: fixed period (not exceeding 120 days)
Alcohol monitoring	
• Twice‐daily breathalysing at police stations • Offender to pay £1 for each breathalyser test	• Alcohol tags • Offenders did not pay for their monitoring
Violations	
• Police to escort the offender to a custody suite or prison • Flash incarceration and prompt appearance before a judge/magistrate who decides on a punishment (including prison)	• Probation service officer supervision • Standard English and Welsh breach processes (first breach = warning; second breach = breach proceedings commence)
Target offenders	
• Anyone convicted of an alcohol‐related crime, but in particular night‐time economy offenders, domestic violence perpetrators and those who drink and drive	• Judicial decision. However, violent individuals, night‐time economy offenders and drink‐drivers were identified by MOPAC as potential targets • Domestic violence perpetrators, dependent drinkers and those with specific medical conditions were excluded

*Sources*: 13, 57, 71, 75, 76, 77, 78, 79, 80, 81, 82, 83. MOPAC = Mayor's Office for Policing and Crime; AAMR = Alcohol Abstinence Monitoring Requirement.

## Policy transfer analysis

Within the mainstream policy transfer literature five main analytical approaches can be detected: process‐centred, comparative, ideational, practice‐based and multi‐level 20. While all these approaches have strengths and weaknesses, it is multi‐level analysis that is arguably the most comprehensive and sophisticated. Scholars associated with this tradition seek to understand the outcomes of policy transfer by examining structure and agency across different levels, ranging from the inter/transnational‐level to the micro‐level. Prominent multi‐level policy transfer models have been generated by Dolowitz & Marsh 21 and Evans & Davies 22. Combined, these models distinguish a number of factors that can facilitate and/or constrain policy borrowing including, *inter alia*, language similarities; institutional and ideological compatibility; economic and technical feasibility; past policies; policy complexity; political leadership; and the influence of networks. Both these models also recognize that policy importation rarely results in photocopying (i.e. the pure replication of a programme). Instead, ‘soft’ (e.g. ideas, attitudes, rhetoric) or ‘hard’ (e.g. tools, practices) elements of non‐indigenous schemes are typically fused with existing domestic programmes and practices to create a locally sensitive policy hybrid.

Following a brief description of methods, the remainder of this paper adopts a multi‐level framework to analyse the factors that enabled and/or hindered processes of international–subnational policy transfer from South Dakota to South London, and that resulted in the design of MOPAC's AAMR Pilot morphing over time. Structure and agency are examined across four dimensions: the global and inter/transnational level; the macro‐state level; the meso‐level; and the micro‐level. Discussion is initially focused on factors that aided and/or obstructed broader transfer processes, before moving to the factors that led to domestic abusers being deemed ineligible for inclusion in the pilot and alcohol tags being utilized as the sole monitoring technology. The factors that led to two core elements of 24/7 Sobriety—offender pays and ‘swift, certain, and fair’ punishment—being abandoned will then be presented.

## Methods

Consistent with an exploratory case study design 23, data were collected to enable the (in)formal events behind the development and implementation of MOPAC's AAMR Pilot to be reconstructed. Two complementary sources of evidence were triangulated using a cross‐checking approach to increase trustworthiness and credibility: elite interviews and documentary materials 24, 25, 26. In total, 25 qualitative time‐line interviews (see 27, 28 were conducted with political, professional, business or administrative elites who were directly involved in, or who were knowledgeable about, the pilot. While anonymity was offered to interviewees, 17 were content to be identified, including: Kit Malthouse (see above); Baroness Finlay of Llandaff; Professor Keith Humphreys (Stanford University); Joe Mitton (Special Adviser, Greater London Authority), Matthew Mitchell (UK Country Manager, Alcohol Monitoring Systems Ltd); Amit Sethi (AAMR Project Manager, MOPAC); and Karyn McCluskey (Director, Scottish Violence Reduction Unit). The primary objective of the interviews was to capture first‐hand insider perspectives concerning the complex processes of policy formation and change 25, 26, 27, 28, 29, 30, 31, 32. Supporting case construction, approximately 200 primary, secondary and tertiary documents were retrieved, including: Acts of Parliament, Bills, Statutory Instruments and Green and White Papers, political manifestos, speeches, evaluation reports, minutes from meetings, letters, newspaper articles, academic publications, websites and blogs.

## Results

Findings indicate that a series of factors facilitated and/or constrained crime and justice policy transfer with regard to MOPAC's AAMR Pilot. As illustrated in Table 2, these factors can be conceptualized within a multi‐level schema.

**Table 2:** Multi‐level facilitating and/or constraining factors.

Global and inter/transnational level	
(F)	New crime control technology
	Alcohol tags were commercially available in the UK
(F)	Globalization
	The internet and transatlantic travel enabled policy tourism within the Greater London Authority
Macro‐state level	
(F)	English and Welsh penal culture and public opinion
	Neoliberal political economy; positive attitude of London residents concerning the notion of enforced alcohol abstinence
(F)	Large prison population
	Incarceration costs were posing a challenge to the state's budget
(F)(C)	Central government agenda
	Alcohol‐related crime had been elevated to the ‘problem’ sphere; localism was a policy initiative; Transforming Rehabilitation was an implementation priority
(C)	Centralization
	Central government is responsible for formulating English and Welsh penal policy. Malthouse and his team were seeking to exercise influence in a policy area beyond their regional remit
(C)	Past policies and legal compatibility
	New primary legislation was required to permit regular alcohol testing and offender pays
(C)	Global financial crisis
	The Coalition Government was delivering an austerity programme—AAMR pilot monies were not available
Meso‐level	
(F)	Mobilization of elite allies
	A cross‐party compulsory sobriety advocacy coalition formed with the objective of securing AAMR legislation
(F)	Influence
	The Greater London Authority is situated within a site of political power (London) and has a high‐profile figurehead (the Mayor of London) who is responsible for a large geographic area
(F)(C)	Political ‘games’
	Parliamentary whipping; policy bargaining; media engagement; breaking promises; stalling; thwarting implementation plans
(F)(C)	Professional, political and media receptivity
	No significant media backlash or opposition from alcohol experts
(C)	Whitehall receptivity and culture
	Aversion to risk and radical policy change; reluctance to introduce new sentences; prejudice concerning innovations that have emerged in the United States; dismissal of the 24/7 Sobriety evidence base; lack of intellectual seduction
Micro‐level	
(F)(C)	Personality traits and qualities of agents of transfer
	Stubbornness; determination; tenacity
(F)(C)	Leadership changes
	Key agents left their positions within the Ministry of Justice and MOPAC
(C)	Ministerial receptivity
	Secretary of State for Justice ➔ legislative territoriality; anti‐localism; cynicism regarding the 24/7 Sobriety cause‐and‐effect model; attitude and values Home Secretary ➔ legislative territoriality; concerns pertaining to the burden that would be placed on the police

F = facilitator; C = constraint; MOPAC = Mayor's Office for Policing and Crime; AAMR = Alcohol Abstinence Monitoring Requirement.

## Domestic violence perpetrators

Evidence suggests that the decision to exclude domestic violence perpetrators from MOPAC's AAMR trial was a product of meso‐level resistance from the UK Violence Against Women and Girls (VAWG) community, and was made after a multi‐agency compulsory sobriety lobbying alliance (see 12) had persuaded the Coalition Government to include AAMR legislation within the *Legal Aid, Sentencing and Punishment of Offenders Act 2012*. To expand, although some VAWG experts had not expressed strong views or had offered contingent support towards Malthouse's aspiration to import enforced alcohol abstinence, others had been expressing hostility towards emulating 24/7 Sobriety in London for quite some time 33, 34, 35, 36. Nevertheless, it was subsequent to statements being made in the House of Lords that suggested that domestic violence specialists overwhelmingly backed the notion of imposing compulsory sobriety on domestic abusers that 13 VAWG organizations issued a Joint Statement conveying their disapproval 37. This interest‐group mobilization led to a meeting being held that was attended by a range of AAMR stakeholders, including central government officials. While not quarrelling with the notion that the severity of an assault can be greater when a perpetrator has consumed alcohol or other substances, VAWG professionals aired multiple objections to MOPAC's intention to include abusers within its AAMR Pilot. A number of these objections are summarized in Table 3, and fall within the themes of messaging, risk and support services.

**Table 3:** Objections articulated by the VAWG community.

Messaging	• Patriarchy is the cause of domestic violence, not alcohol. Sobriety will not address the root causes of a perpetrator's behaviour • Perpetrators may blame alcohol for their actions rather than taking responsibility for their own thoughts and choices
Risk	• A victim may choose not to adopt particular measures or flee from a life‐threatening situation due to the mistaken belief that they are safer because their partner is not consuming alcohol • The AAMR will simply produce a ‘sober abuser’ who is still capable of inflicting physical, emotional, psychological, sexual and financial abuse • Perpetrators may seek revenge on victims for their sentence
Support services	• Long waiting‐lists mean that perpetrator programmes are unlikely to be delivered in tandem with an AAMR

*Sources*: 34, 35, 36, 37, 38, 39, 40, 84. MOPAC = Mayor's Office for Policing and Crime; AAMR = Alcohol Abstinence Monitoring Requirement; VAWG = Violence Against Women and Girls.

In siding with the VAWG community and exercising its power to dictate English and Welsh penal policy, the government subsequently announced that MOPAC's pilot could not include domestic violence perpetrators. Disappointment aside, Malthouse was decidedly pragmatic when reflecting on the matter when interviewed in 2015, his attitude being that compromises had to be made to experiment with compulsory sobriety in London, and that positive evaluation results would bolster future attempts to mimic the South Dakota model more closely 38.

Well, I was disappointed […] we came to the view that if you pitch high and come in lower, you come in low enough that you still think that the structure of the scheme will prove its efficacy and that over time people will then start accepting moving towards the full model’—interviewee: Kit Malthouse.

Interestingly, Malthouse's prediction was relatively accurate. Following what a government junior minister described as ‘very encouraging’ results, the Cameron Government (2015–16) formally extended MOPAC's AAMR trial for 6 months in July 2015 while discussions took place about the pilot's longer‐term future 39, 40, 41, 42, 43, 44. MOPAC later announced that its compulsory sobriety scheme would be rolled out in phases across the whole of London from April 2016, and that domestic abusers would be eligible to receive an AAMR 45, 46, 47, 48, 49. This pilot concluded in summer 2018. In addition, in April 2017 permission to launch a 2‐year AAMR trial in the north of England was granted by the first May ministry (2016–17) 50. This pilot is funded by the Police and Crime Commissioners for each of the regions involved and, notably, includes domestic violence perpetrators 51, 52, 53.

## Alcohol tags

Several considerations influenced MOPAC's decision to use alcohol tags rather than twice‐daily breathalysing to ensure AAMR compliance. The first centred on macro‐domestic financial and political constraints. MOPAC officers were concerned about the resource implications of breathalyser testing, especially as they were operating within a context of shrinking public services and probation upheaval in the form of Transforming Rehabilitation 54, 55, 56. Indeed, with regard to the latter, MOPAC officers were reluctant to place additional strain on probation staff due to the impending split of community service provision between the National Probation Service and private‐sector Commercial Rehabilitation Companies. A second consideration centred on intrusion, with MOPAC officials fearing the disruption that frequently travelling to a testing site could mount with respect to an offender's work and/or study routine 57. A third consideration centred on enforcement. Given that alcohol tags permit continuous monitoring of alcohol consumption, MOPAC agents were attracted to the speed in which an AAMR infraction could be identified 57.

Lastly, evidence suggests that MOPAC's preference for alcohol tags was an outcome of international and subnational policy‐orientated learning. MOPAC representatives who were responsible for erecting London's AAMR Pilot tuned‐in to the voices of those who had attained experience of deploying alcohol tags in the United Kingdom and/or the United States. In relation to the subnational–subnational interaction that occurred between UK‐based individuals, MOPAC officials engaged with a district judge who endorsed alcohol tags, having used them efficaciously within child protection cases that were brought to a Family Drug and Alcohol Court in London 58. In addition, MOPAC agents communicated with Karyn McCluskey, a Director of the high‐profile Scottish Violence Reduction Unit who had trialled alcohol tags under an initiative branded ‘Project Pegasus’ and who had become a firm advocate for their deployment across the United Kingdom 35, 59.

I gave everything to London, all of my papers, I gave hundreds of files […] I said ‘don't reinvent it; here is how to do [the pilot]’. I spoke to all the policy people down at the Mayor's Office around how they should it. We all succeed together; it is not individual—interviewee: Karyn McCluskey.

With regard to international lesson‐drawing, a week‐long fact‐finding visit to the United States by a MOPAC Special Adviser played a role in the abandonment of breathalysers. This adviser accepted an invitation forwarded by the National Association of Drug Court Professionals (NADCP) to attend its Annual Training Conference in Washington, DC 39, 60, 61, 62. While in the United States, they also visited New York and several locations in Michigan, where they met with criminal justice professionals, academics, programme evaluators and private‐sector providers of alcohol tags. Upon returning to London, they made the case for employing alcohol tags 39, 56. Indeed, they reportedly informed their colleagues that almost every subnational agent that they had spoken to in the United States had maintained that their jurisdiction was using alcohol tags or were moving towards them not only because of the reliability of the technology and its ability to monitor compliance around the clock, but also because police time was being wasted chasing those who may not have consumed alcohol yet who had skipped a breath test 39. Their advice was heeded 39, 56.

## Offender pays and ‘swift, certain and fair’ justice

Findings suggest that the ‘non‐transfer’ 20 of offender pays was a consequence of macro‐state level legal incompatibility and meso‐level Whitehall reluctance. No penal mechanism existed in England and Wales to compel an offender to pay the costs of delivering a non‐financial penalty 63. In addition, Ministers were disinclined to legislate in favour of a daily fine model, as civil servants within the Ministry of Justice were concerned that a precedent would be set whereby offenders could pay their fines gradually and in small amounts, hence increasing administrative pressures on the criminal justice system 64, 65.

Interestingly, US–UK legal discordancy was also cited as a reason as to why the Government would not permit those who breached their AAMR to be ‘flash incarcerated’ as part of MOPAC's pilot. Ministers stated that due to *habeas corpus* the European Court of Human Rights could object to offenders who breach their AAMR being detained without trial 66. The upshot was that South Dakota's ‘skip or fail = jail’ sequence could not be replicated. Instead, the AAMR breach process had to align with that of any other Community or Suspended Sentence Order as enshrined in the *Criminal Justice Act 2003* 67, 68, 69. Unless a breach was considered serious, the standard procedure was thus ‘query + no reasonable excuse = first warning; query + no reasonable excuse = breach’. Effectively, the offender could crack once without penalty.

In reaction to this transfer obstruction, MOPAC agents did strive to incorporate the spirit of the ‘swift, certain and fair’ philosophy into their pilot in a manner that was consistent with the English and Welsh criminal justice framework. A bespoke non‐compliance notification process was implemented that entailed staff employed by MOPAC's alcohol tag provider immediately contacting an offender following a suspected breach 70. It should be noted, however, that swift and certain acknowledgement of breach is not analogous with ‘swift, certain and fair’ justice. Given that completion of the AAMR breach process took up to 25 days, offenders had to wait to reappear in front of a magistrate or judge who, in turn, selected a punishment from a range of modest options 70. In essence, offenders were told: ‘If you do something that you shouldn't we will catch you, and if you don't have a reasonable excuse and satisfactory evidence you will definitely be punished at some point in the future, though when and how exactly remain hazy’.

## Discussion and conclusions

This is the first paper, to the author's knowledge, to directly examine the multi‐level factors that facilitate and/or constrain international–subnational crime and justice policy transfer. It is also the first to provide a synopsis of the formulation of MOPAC's AAMR Pilot, which itself was the first trial in Europe to combine enforced alcohol abstinence with alcohol tagging 71. Results confirm that a series of inter/transnational‐, macro‐domestic‐, meso‐ and micro‐level factors served to enable and/or limit transfer processes. They included: European policy harmonization; technological innovation; existing English and Welsh criminal justice legislation; political–economic climate; policy‐orientated learning; and agent receptivity and mobilization. Ultimately, the architecture of MOPAC's scheme was a ‘synthesis’ 72 that combined ‘soft’ (i.e. ideas, goals, principles and vocabulary) and ‘hard’ (i.e. technology, practices) elements of the South Dakota 24/7 Sobriety Project with existing English and Welsh penal infrastructure, institutions and regulations. This outcome is far from unexpected. As discussed above, existing policy transfer models have highlighted that ‘cut‐and‐paste’ policy transfer is uncommon and that contextual adaptation is the norm.

Nonetheless, this case study reveals a number of important findings, not least that international–subnational crime control policy transfer is a highly complex and contested form of policy development, even for those operating within a powerful and influential jurisdiction such as Greater London. Years elapsed between Malthouse's attendance at the OPPF and the implementation of a ‘Londonized’ version of 24/7 Sobriety. Although the findings of a single case should not be used to predict future events 26, such policy blockage suggests that, like their national‐level counterparts, subnational policymakers may not only need to be wary of seeking to import a ready‐made overseas innovation to ‘quickly fix’ a local problem, but should also be primed to re‐imagine and mutate their transferred policy idea in response to domestic resistance and structural incompatibility. Whether such policy morphing was detrimental to the ‘success’ of MOPAC's pilot is a moot point. Further evaluation findings are required before any robust claims can be made concerning the efficacy of London's compulsory sobriety trial. These findings are due to be published by MOPAC later this year. Nevertheless, given that nearly 1200 AAMRs had been imposed in London by March 2018, and that a compliance rate of more than 90% was attained, questions concerning the necessity of faithfully emulating 24/7 Sobriety's cause‐and‐effect model are certainly being raised 73.

## Declaration of interests

None.

## References

[1] Evans M. , Barakat S. Post‐war reconstruction, policy transfer and the World Bank: the case of Afghanistan's National Solidarity Programme. Policy Stud 2012; 33: 531–565.

[2] Nellis M. Law and order: the electronic monitoring of offenders In: Dolowitz D. P., Hulme R., Nellis M., O'Neill F., editors. Policy Transfer and British Social Policy. Buckingham: Open University Press; 2000; 98–117.

[3] Newburn T. Police and Crime Commissioners: the Americanization of policing or a very British reform? Int J Law Crime Justice 2012; 40: 31–46.

[4] Jones T. , Newburn T. Policy convergence, politics and comparative penal reform: sex offender notification schemes in the USA and UK. Punishment Soc 2013; 15: 439–467.

[5] Jones T. , Newburn T. Policy Transfer and Criminal Justice. Maidenhead: Open University Press; 2007.

[6] Schachter H. L. Bail interviewing: a case of cross‐national policy transfer. Int Crim Justice Rev 1991; 1: 21–34.

[7] Stenson K. , Edwards A. Policy transfer in local crime control: beyond naïve emulation In: Newburn T., Sparks R., editors. Criminal Justice and Political Cultures. Cullompton: Willan; 2004; 209–233.

[8] Edwards A. , Hughes G. Editorial. Theor Criminol 2005; 9: 259–263.

[9] Mawby R. I. A letter from Cyprus: migration, policy transference and the piloting of neighbourhood watch. Crime Prev Commun Saf 2011; 13: 148–152.

[10] Wincup E. Understanding Crime and Social Policy. Bristol: Policy Press; 2013.

[11] Gavens L. , Holmes J. , Buykx P. , de Vocht F. , Egan M. , Grace D. *et al* Processes of local alcohol policy‐making in England. Does the theory of policy transfer provide useful insights into public health decision making? Health & Place 2017 Available at: https://www.sciencedirect.com/science/article/pii/S1353829216302428 (accessed 10 April 2019). 10.1016/j.healthplace.2017.05.016 28622872

[12] Bainbridge L. Exploring ‘international–subnational’ crime and justice policy transfer: the case of MOPAC's Alcohol Abstinence Monitoring Requirement Pilot. PhD Thesis, University of York; 2017. Available at: http://etheses.whiterose.ac.uk/19793/

[13] Metropolitan Police Authority Web Archive . Transcript of the meeting of the Metropolitan Police Authority. Kew: The National Archives; 2010.

[14] Longstaff, A. Report of the eighth Oxford Policing Policy Forum. Oxford: All Souls College, Oxford; 2010.

[15] Caulkins J. P. Policing drugs and alcohol: is harm reduction the way forward? [PowerPoint Presentation]. Presented at the Oxford Policing Policy Forum, All Souls College, Oxford, 8 February; 2010.

[16] Long L. The 24/7 Sobriety Project. Public Lawyer 2009; 17: 2–5.

[17] Kilmer B. , Humphreys K. Losing your ‘license to drink’: the radical South Dakota approach to heavy drinkers who threaten public safety. Brown J World Aff 2013; 20: 267–277.

[18] Long, L. Overview of the first 24/7 program in South Dakota. In: The National 24/7 Sobriety Advisory Council. 24/7 Sobriety Program: essential elements and best practices. Sioux Falls, SD: The National 24/7 Sobriety Advisory Council; 2016.

[19] Voas R. B. , DuPont R. L. , Talpins S. K. , Shea C. L. Towards a national model for managing impaired driving offenders. Addiction 2011; 106: 1221–1227. 21205054 10.1111/j.1360-0443.2010.03339.x

[20] Evans M. Understanding policy transfer in the competition state In: Fenwick J., McMillan J., editors. Public Management in the Postmodern Era: Challenges and Prospects. Cheltenham: Edward Elgar Publishing; 2010; 64–96.

[21] Dolowitz D. P. , Marsh D. Who learns what from whom? A review of the policy transfer literature. Polit Stud 1996; 44: 343–357.

[22] Evans M. , Davies J. Understanding policy transfer: a multi‐level, multi‐disciplinary perspective. Public Adm 1999; 77: 361–385.

[23] Yin R. K. Case Study Research: Design and Methods. London: Sage Publications; 2014.

[24] Guba E. G. Criteria for assessing the trustworthiness of naturalistic inquiries. Educ Commun Technol 1981; 29: 75–91.

[25] Richards D. Elite interviewing: approaches and pitfalls. Politics 1996; 16: 199–204.

[26] Tracy S. H. Qualitative quality: eight ‘big‐tent’ criteria for excellent qualitative research. Qual Inq 2010; 16: 837–851.

[27] Berends L. Embracing the visual: using timelines with in‐depth interviews on substance use and treatment. Qual Rep 2011; 16: 1–9.

[28] Kolar K. , Ahmad F. , Chan L. , Erickson P. G. Timeline mapping in qualitative interviews: a study of resilience with marginalized groups. Int J Qual Methods 2015; 14: 13–32.

[29] Koemans M. The war on street ‘terror’. Why tackle anti‐social behaviour? Crime Law Soc Chang 2010; 53: 477–491.

[30] Leech B. L. Interview methods in political science. Polit Sci Polit 2002; 35: 663–664.

[31] Lilleker D. G. Interviewing the political elite: navigating a potential minefield. Politics 2003; 23: 207–214.

[32] Xenakis S. , Ivanov K. Does hypocrisy matter? National reputational damage and British anti‐corruption mentoring in the Balkans. Crit Criminol 2016; 25: 433–453.

[33] *Hansard* HL Deb. v.736 c802–815; 2012.

[34] Interviewee: Joe Mitton (Special Adviser, Office of the Mayor of London, 2010**–**2012).

[35] Interviewee: Anonymous—1.

[36] Interviewee: Anonymous—2.

[37] AVA, Barnet Domestic Violence Support Services, Eaves, Housing For Women, Imkaan, Nia Project. Refuge, Respect, Rights of Women, Stand Together Against Domestic Violence, Solace Women's Aid, National Women's Aid and University of Bedfordshire. *Joint Statement: Compulsory Alcohol Sobriety Orders and Domestic Violence*; 2012.

[38] Interviewee: Kit Malthouse (London's Deputy Mayor for Policing, 2008–2012).

[39] Interviewee: MOPAC Representative 1.

[40] Violence Against Women and Girls Panel Meeting. *Minutes* 14 March; 2012 Available at: https://www.london.gov.uk/sites/default/files/vawg_minutes_14_march_2012.pdf (accessed 27 March 2019) (Archived at http://www.webcitation.org/77BqBEL2m on 27 March 2019).

[41] Alcohol Policy UK. *Main party manifestos quiet on alcohol policy and minimum pricing* 2015 Available at: https://www.alcoholpolicy.net/2015/04/main-party-manifestos-quiet-on-alcohol-policy-and-minimum-pricing-.html (accessed 20 February 2019) (Archived at http://www.webcitation.org/76KUtRB7R on 20 February 2019).

[42] BBC News . ‘*Sobriety tag’ scheme to be extended for six months* 2015 Available at: https://www.bbc.co.uk/news/uk-england-london-33675874 (accessed 20 February 2019) (Archived at http://www.webcitation.org/76KWTnJ8j on 20 February 2019).

[43] Crerar P. Sobriety tags on London's worst lager louts will alert probation officers if they drink too much. *Evening Standard*, 2015 Available at: https://www.standard.co.uk/news/london/tags-on-londons-worst-lager-louts-will-alert-probation-officers-if-they-drink-too-much-10418285.html (accessed 20 February 2019) (Archived at http://www.webcitation.org/76KWm8vxh on 20 February 2019).

[44] Mayor's Office for Policing and Crime. *Request for DMPC Decision, DMPCD 2015 142* London: Mayor's Office for Policing and Crime; 2015.

[45] Sethi A. , Mitchell M. *London's Compulsory Sobriety Pilot* [PowerPoint Presentation]; 2016.

[46] *Legal Aid, Sentencing and Punishment of Offenders Act 2012 (Alcohol Abstinence Monitoring Requirement, Piloting Order 2015)*, SI 2015/1480 c.86. Available at: http://www.legislation.gov.uk/uksi/2015/1480/pdfs/uksi_20151480_en.pdf (accessed 27 March 2019) (Archived at http://www.webcitation.org/77BsoyGZy on 27 March 2019).

[47] Mayor of London. *News release: ‘Sobriety tags’ rolled out across London* 2016 Available at: https://www.london.gov.uk/press-releases/mayoral/crackdown-against-alcohol-related-crime (accessed 20 February 2019) (Archived at http://www.webcitation.org/76KTdHV3P on 20 February 2019).

[48] BBC News . *‘Sobriety tags’ rollout as 92% comply in pilot schemes* 2016 Available at: https://www.bbc.co.uk/news/uk-england-london-35660946 (accessed 20 February 2019) (Archived at http://www.webcitation.org/76KT859Eu on 20 February 2019).

[49] Malthouse K. *Sobriety tags to be rolled out across all London courts* 2016 Available at: http://kitmalthouse.com/2016/sobriety-tags-to-be-rolled-out-across-all-london-courts/ (accessed 20 February 2019) (Archived at http://www.webcitation.org/76KS3SUGI on 20 February 2019).

[50] *Legal Aid, Sentencing and Punishment of Offenders Act 2012 (Alcohol Abstinence Monitoring Requirement, Piloting Order 2017)*, SI 2017/525 c.48. Available at: http://www.legislation.gov.uk/uksi/2017/525/made/data.htm (accessed 27 March 2019) (Archived at http://www.webcitation.org/77BsL1FSA on 27 March 2019).

[51] Humberside, Lincolnshire and North Yorkshire Community Rehabilitation Company. *Pilot scheme launched in region to reduce alcohol‐related crime* 2017 Available at: http://www.hlnycrc.co.uk/pilot-scheme-launched-humberside-reduce-alcohol-related-crime/ (accessed 20 February 2019) (Archived at http://www.webcitation.org/76KRqRqWo on 20 February 2019) .

[52] Humberside Police and Crime Commissioner. *Pilot scheme launched to reduce alcohol‐related crime* 2017 Available at: https://www.humberside-pcc.gov.uk/News/News-Archive/2017/Pilot-Scheme-launched-to-reduce-alcohol-related-crime.aspx (accessed 10 April 2019) (Archived at http://www.webcitation.org/77WjeAJZ6 on 10 April 2019).

[53] Lincolnshire Police and Crime Commissioner. *Sobriety tags being launched in Lincolnshire* 2017 Available at: https://lincolnshire-pcc.gov.uk/news-archive/2017/sobriety-tags/ (accessed 20 February 2019) (Archived at http://www.webcitation.org/76KQreBRC on 20 February 2019).

[54] Interviewee: Keith Humphreys (Professor, Stanford University).

[55] Interviewee: Matthew Mitchell (Alcohol Monitoring Systems Ltd).

[56] Interviewee: MOPAC Representative 2.

[57] Lamb L. Alcohol Abstinence and Monitoring (AAMR)—MOPAC London Pilot. Privacy Impact Assessment Report. London: Ministry of Justice; 2013.

[58] Vallance C. Are ankle tags the answer for tackling parents with drink problems*?* *BBC News* 2013 Available at: https://www.bbc.co.uk/news/uk-21882189 (accessed 20 February 2019) (Archived at http://www.webcitation.org/76KQXKtid on 20 February 2019).

[59] Interviewee: Karyn McCluskey (Director, Scottish Violence Reduction Unit, 2005–2016).

[60] National Association of Drug Court Professionals . *Future and past conferences*; n.d. Available at: https://nadcpconference.org/future-and-past-conferences/ (accessed 20 February 2019) (Archived at http://www.webcitation.org/76KOawnOu on 20 February 2019).

[61] Chambers M. , Davis R. , McLeod C. Power down. A plan for a cheaper, more effective justice system. Policy Exchange 2013; 6: 169–191.

[62] Interviewee: Charlotte McLeod (Crime and Justice Research Fellow, Policy Exchange, 2012–2014).

[63] Hansard HL Deb. v.735 c187–201, 2012 Available at: https://publications.parliament.uk/pa/ld201212/ldhansrd/text/120207-0001.htm#12020769000417 (accessed 10 April 2019) (Archived at http://www.webcitation.org/77WpowYqv on 10 April 2019).

[64] Interviewee: Baroness Finlay of Llandaff. 26867342

[65] Interviewee: Anonymous—3.

[66] *Hansard* HL Deb. v.728 c436–450, 2011 Available at: https://publications.parliament.uk/pa/ld201011/ldhansrd/text/110609-0002.htm (accessed 10 April 2019) (Archived at http://www.webcitation.org/77WjuBEeJ on 10 April 2019).

[67] Interviewee: Amit Sethi (London AAMR Project Manager, 2014–2017).

[68] Interviewee: Anonymous—4.

[69] Interviewee: Anonymous—5.

[70] Mayor's Office for Policing and Crime Alcohol Abstinence Monitoring Requirement, Toolkit. Version 2. London: Mayor's Office for Policing and Crime; 2014 Available at: https://www.london.gov.uk/sites/default/files/aamr_toolkit_october_2014_v2.pdf (accessed 27 March 2019) (Archived at http://www.webcitation.org/77BqHNBCl on 27 March 2019).

[71] Pepper M. , Dawson P. Alcohol Abstinence Monitoring Requirement. A Process Review of the Proof of Concept Pilot. London: MOPAC; 2016.

[72] Rose R. Learning From Comparative Public Policy: A Practical Guide. London: Routledge; 2005.

[73] Bainbridge L. Sobriety tags: how alcohol monitoring could become the new norm. The Conversation, 2018 Available at: https://theconversation.com/sobriety-tags-how-alcohol-monitoring-could-become-the-new-norm-98975 (accessed 20 February 2019) (Archived at http://www.webcitation.org/76KNX4ArD on 20 February 2019).

[74] Mayor's Office for Policing and Crime . *Request for DMPC Decision, PCD 358* London: Mayor's Office for Policing and Crime; 2018 Available at: https://www.london.gov.uk/what-we-do/mayors-office-policing-and-crime-mopac/governance-and-decision-making/mopac-decisions-0/alcohol-abstinence-monitoring-requirement-aamr-pan-london-extension (accessed 27 March 2019) (Archived at http://www.webcitation.org/77BrpvsQk on 27 March 2019).

[75] BBC News . *24/7 ‘sobriety’ or jail scheme may be piloted in London*; 2010. Archived at: https://www.bbc.co.uk/news/uk-england-london-11633677 (accessed 20 February 2019) (Archived at http://www.webcitation.org/76Ka8pPgg on 20 February 2019).

[76] BBC Radio 4 . *Today*; 2010.

[77] Express. *UK trial for sobriety scheme urged*; 2010. Available at: https://www.express.co.uk/news/uk/207866/UK-trial-for-sobriety-scheme-urged (accessed 20 February 2019) (Archived at http://www.webcitation.org/76KaJ8qxb on 20 February 2019).

[78] Malthouse K. The South Dakota cure for our drink problem *. The Times*, 2010 Available at: http://kitmalthouse.com/2010/the-south-dakota-cure-for-our-drink-problem/ (accessed 20 February 2019) (Archived at http://www.webcitation.org/76KZcJVAU on 20 February 2019).

[79] Mulholland H. Boris Johnson calls for ‘24/7 sobriety’ scheme to combat drink‐fuelled crime. *Guardian*, 2010 Available at: https://www.theguardian.com/politics/2010/oct/27/boris-johnson-london-24-7-sobriety-south-dakota (accessed 20 February 2019) (Archived at http://www.webcitation.org/76KZQ4TdI on 20 February 2019).

[80] Roberts L. Problem drinkers could face compulsory alcohol tests. *Telegraph*, 2010 Available at: https://www.telegraph.co.uk/news/uknews/law-and-order/8089259/Problem-drinkers-could-face-compulsory-alcohol-tests.html (accessed 20 February 2019) (Archived at http://www.webcitation.org/76KZ1zCDZ on 20 February 2019).

[81] London Assembly . *24th Mayor's Report to the Assembly*; 2014.

[82] Mayor's Office for Policing and Crime Leaflet—Compulsory Sobriety Pilot. London: MOPAC; n.d.

[83] Mayor's Office for Policing and Crime . Functional Specification. Alcohol Abstinence Monitoring Requirement Request for Quote. London: MOPAC; n.d.

[84] Humphreys K. Alcohol as a non‐exculpatory facilitator of violence against women. *The Reality‐Based Community* 2012 Available at: http://www.samefacts.com/2012/11/drug-policy/alcohol-as-a-non-exculpatory-facilitator-of-violence-against-women/ (accessed 20 February 2019) (Archived at http://www.webcitation.org/76KUZtSVQ on 20 February 2019).

